# Cardiovascular magnetic resonance tagging of the right ventricular free wall for the assessment of long axis myocardial function in congenital heart disease

**DOI:** 10.1186/1532-429X-13-80

**Published:** 2011-12-14

**Authors:** Sylvia SM Chen, Jennifer Keegan, Andrew W Dowsey, Tevfik Ismail, Ricardo Wage, Wei Li, Guang-Zhong Yang, David N Firmin, Philip J Kilner

**Affiliations:** 1 Royal Brompton Hospital, Sydney Street, London SW3 6NP, UK; 2 Imperial College, South Kensington Campus, London SW7 2AZ, UK

**Keywords:** Right Ventricle, Ejection Fraction, Cardiac MRI, Tagging, Congenital Heart Disease, Reproducibility

## Abstract

**Background:**

Right ventricular ejection fraction (RV-EF) has traditionally been used to measure and compare RV function serially over time, but may be a relatively insensitive marker of change in RV myocardial contractile function. We developed a cardiovascular magnetic resonance (CMR) tagging-based technique with a view to rapid and reproducible measurement of RV long axis function and applied it in patients with congenital heart disease.

**Methods:**

We studied 84 patients: 56 with repaired Tetralogy of Fallot (rTOF); 28 with atrial septal defect (ASD): 13 with and 15 without pulmonary hypertension (RV pressure > 40 mmHG by echocardiography). For comparison, 20 healthy controls were studied. CMR acquisitions included an anatomically defined four chamber cine followed by a cine gradient echo-planar sequence in the same plane with a labelling pre-pulse giving a tag line across the basal myocardium. RV tag displacement was measured with automated registration and tracking of the tag line together with standard measurement of RV-EF.

**Results:**

Mean RV displacement was higher in the control (26 ± 3 mm) than in rTOF (16 ± 4 mm) and ASD with pulmonary hypertension (18 ± 3 mm) groups, but lower than in the ASD group without (30 ± 4 mm), P < 0.001. The technique was reproducible with inter-study bias ± 95% limits of agreement of 0.7 ± 2.7 mm. While RV-EF was lower in rTOF than in controls (49 ± 9% versus 57 ± 6%, P < 0.001), it did not differ between either ASD group and controls.

**Conclusions:**

Measurements of RV long axis displacement by CMR tagging showed more differences between the groups studied than did RV-EF, and was reproducible, quick and easy to apply. Further work is needed to assess its potential use for the detection of longitudinal changes in RV myocardial function.

## Background

The assessment of RV function is particularly important in the setting of congenital heart disease where detecting deterioration of RV function by cardiovascular magnetic resonance (CMR) may be considered an indication for operative intervention [1,2]. However, there is currently no established objective CMR method of evaluating RV long-axis function. CMR tagging techniques have been used to assess long axis function of the LV [3], and of the RV in healthy volunteers [4,5]. However, the acquisition of tagged data covering all three dimensions and recording all three directional components of displacement is time-consuming, requires sophisticated post-processing [4] and remains unlikely to be adopted clinically. A less complex method of acquiring RV tagged images exists, but post-processing time is again long and not straight forward [5].

We therefore developed a technique designed to expedite the acquisition and analysis of data on RV long axis function. A linear tag was oriented perpendicular to the basal free wall, allowing a simple automated measurement of its apically directed displacement. We hypothesized that this technique would be reproducible and could differentiate between populations with normal RVs and those with pressure and/or volume loading.

## Methods

### Study population

Eighty four patients undergoing clinical CMR at Royal Brompton Hospital with either previously repaired tetralogy of Fallot (rTOF) with pulmonary regurgitation (PR) (n = 56), or unoperated atrial septal defect (ASD) with pulmonary hypertension (PHT) (n = 13) or without PHT (n = 15) were recruited prospectively, PHT being defined as an estimated RV systolic pressure > 40 mmHg derived from the peak tricuspid regurgitant jet velocity plus right atrial pressure estimated (from the inferior vena cavae calibre at hepatic level) on echocardiography [6]. Twenty healthy control subjects (age 41 ± 12 years, 11 females and 9 males) also underwent assessment for comparison with the patient population. In addition, in this control group, a subset of 10 of the 20 volunteers was brought out of the magnet between CMR acquisition by two independent operators in order to assess the inter-study variability of both image acquisition and analysis. Written informed consent was obtained from all subjects and the study was approved by our institutional Ethics Committee.

### CMR protocol

**Figure 1:**
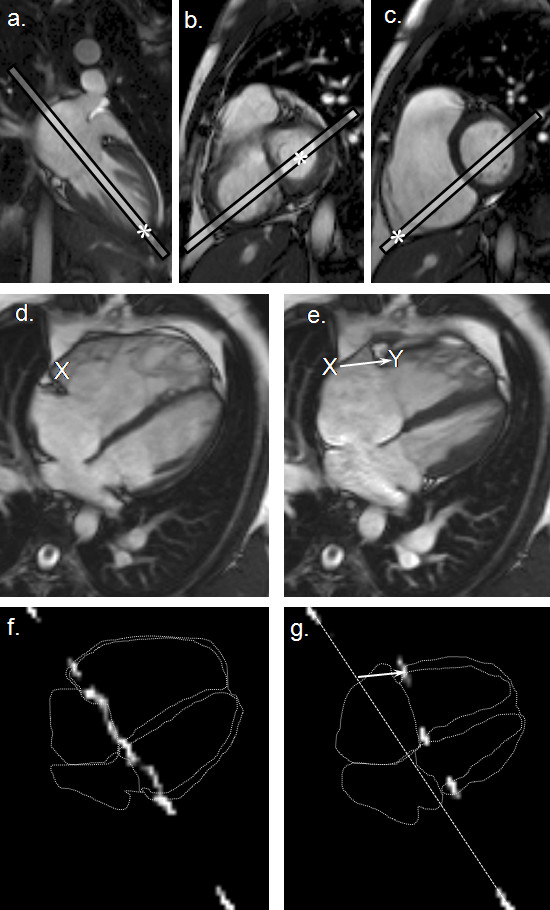
**Alignment of the 4-chamber cine and the tagging by CMR**. Three points marked (*) in the scout images (a-c) were used to define the 4-chamber image plane (d and e). The post-subtraction CMR tissue tag images at end-diastole (f) and end-systole (g) are shown superimposed on outlines traced from the corresponding 4-chamber cine frames. The white arrow in panel e indicates the systolic displacement of the RV free wall tag relative to a dotted line that has been drawn between the motionless tagged regions of the chest wall. For manual measurement of RV basal free wall displacement, by using CMRtools, a marker (X) was placed in the RV free wall 5 mm from the atrio-ventricular groove at end-diastole (e) and copied to the end-systolic frame (f), where the white arrow indicates the displacement measured (X→Y).

CMR was performed on a 1.5T scanner (Siemens Sonata or Siemens Avanto, Siemens Medical Solutions, Erlangen, Germany) using an 8-element phased-array receiver coil. Subjects were positioned supine in the magnet and scout images obtained. A routine set of LV and RV short-axis cines, 7mm slice thickness, were acquired at 10mm intervals from base to apex using a breath-hold retrospective vector cardiography-gated balanced steady state free precession (SSFP) gradient echo sequence, and volumetric analysis was performed using CMRtools (Cardiovascular Imaging Solutions, UK) [7]. A 4-chamber SSFP cine was acquired by aligning through the apex of the LV in vertical long axis cine, the mid-point of the mitral valve in a basal short-axis cine, and the point of angulation of the RV free wall adjacent to the cardiophrenic angle (Figures 1a-c). This was followed by a breath-hold cine tagged acquisition in the same 4-chamber view (Figures 1d-e). Briefly, a selective and non-selective 90° pulse pair was used to tag a plane passing through the basal RV myocardium and perpendicular to the length of the basal free wall, 5 mm from the atrio-ventricular groove in an orientation equivalent to a ventricular short-axis slice (Figure 1d-e) [8]. Two datasets were acquired in the same breath-hold, with the phase of the selective component of the 90° pulse pair being reversed between the two. Complex subtraction of these two datasets resulted in clear visualization of the tagged region whilst all other material was suppressed (Figures 1f-g). The sequence parameters were as follows: tag thickness 7 mm, slice thickness 15 mm, spatial resolution 3.1 mm × 2.4 mm, temporal resolution 30 ms and a breath-hold duration of 18 cardiac cycles. The setting up and acquisition of the tagged image took under 2 minutes.

The tagged images were analysed automatically once the user had located the line joining the tagged RV free wall and septal myocardium in the first frame of the cardiac cycle [9]. Multi-resolution image registration was used for non-rigid motion estimation of the selected tag points through the cardiac cycle with cubic interpolation between time-points [9]. The result was an output text file showing the displacement of the tagged myocardium through the cardiac cycle. Complete analysis of a tagged dataset typically took 1.5 minutes. Peak systolic myocardial displacement measurements were used for analysis in this study.

### Reproducibility of Image Acquisition and Analysis of Tag Displacement

In a randomly selected subset of twenty four studies (2 controls and 22 patients), measurements of RV basal free wall displacements were made by 2 independent experienced observers (observers 1 and 2) both manually from the CMR 4-chamber cine acquisition (Figures 1d-e), and from the tag acquisition using the automated analysis software. Each method (manual and automated) was performed twice for each subject by both observers on separate occasions.

To assess the inter-study reproducibility of tagged myocardial image acquisition and analysis, 10 control subjects were scanned by two independent operators. Each subject underwent two completely separate scans, one by each operator. The subject was positioned in the scanner, images acquired, and subjects taken out of the scanner by the first operator for the initial scan. Then, the second operator repositioned the patient in the scanner and acquired images in the second scan. Both sets of images were analysed using the automated software by operator 1, and once more by the same operator at a later date.

### Statistical Analysis

Statistical analysis was performed using SPSS (V.19.1, SPSS Inc, Chicago, Illinois, USA). Data is expressed as mean ± SD and as frequencies for categorical data. Normality of all data distributions in the patient and control groups was tested using the Kolmogorov-Smirnov method. The intra- and inter-observer reproducibility of CMR tagging measurements and inter-study reproducibility were assessed using the method of Bland and Altman [10]. RV and LV myocardial displacement comparisons between groups were performed using one way analysis of variance (ANOVA) followed by post-hoc paired t-testing with appropriate correction for multiple comparisons after Levene's test for uniformity of variance. In all cases, a two-tailed value of P < 0.05 was considered statistically significant. The degree of correlation between RV-displacement and RV-ejection fraction was assessed using the Pearson product moment coefficient correlation. The intra-class coefficient of correlation was also used to assess the degree of agreement for reproducibility data.

## Results

### Study population

A total of 84 patients were studied: 56 with rTOF, 15 with unoperated ASD but no PHT, and 13 with ASD and PHT (Table 1). In the rTOF group, two patients had moderate RV outflow tract stenosis and two had moderate right pulmonary artery stenosis, but other than this, the surgically repaired cohort did not have any significant associated structural heart disease (Table 1). There was only 1 patient with mild tricuspid regurgitation. Similarly, the ASD groups were free of any other cardiac anomalies.

**Table 1:** Baseline Clinical and Demographic Characteristics

**Overall patient population**	
Male, n (%)	34/84 (40)
Age, years ( ± SD)	34 ± 15
**Repaired TOF with pulmonary regurgitation**	
Male, n (%)	26 (46)
Age, years ( ± SD)	27 ± 10
Age at TOF Repair, years (± SD)	4 ± 4
Pulmonary Regurgitation Fraction, % (± SD)	36 ± 12
Length of Akinetic Region in the RV Outflow Tract, mm (± SD)	34 ± 16
Other Cardiac Defects or Co-morbidities:	
- RV outflow tract stenosis, n	8 mild, 2 moderate
- Right pulmonary artery stenosis, n	1 mild, 2 moderate
- Left pulmonary artery stenosis, n	1 mild
- Tricuspid regurgitation, n	1 mild
**ASD without pulmonary hypertension**	
Male, n (%)	5 (33)
Age, years ( ± SD)	36 ± 14
Shunt size, Q_P_:Q_S_	2 ± 0.6
**ASD with pulmonary hypertension**	
Male, n (%)	3 (23)
Age, years (± SD)	54 ± 16
Peak Pulmonary Artery Systolic Pressure, mmHg ( ± SD)	85 ± 25 mmHg
Shunt size, Q_P_:Q_S_	2.3 ± 0.6
Other Cardiac Defects or Co-morbidities	None

There was no significant difference between the groups with respect to sex (= 0.491), however, the rTOF group tended to be younger relative to the control (p = 0.002) and ASD with PHT groups (p = 0.004). The indexed LV end diastolic volume in the ASD pulmonary hypertension group was significantly lower than in the control group (p = 0.006). In addition, the indexed LV mass was significantly lower in the ASD pulmonary hypertension group relative to rTOF group (p = 0.003); however, there was no significant difference relative to the control group (p = 1.00, Table 2).

**Table 2:** CMR findings for the four groups (significant differences in bold, p < 0.05)

Characteristic	rTOF (n = 56)	ASD (n = 15)	ASD+PHT (n = 13)	Control (n = 20)	P (rToF & control)	P (ASD & control)	P (ASD+PHT & control
Male, n (%)	26 (46)	5 (33)	3 (23)	9 (45)	0.889	0.476	0.135
Age, years ± SD	27 ± 10	36 ± 14	54 ± 16	41 ± 12	**0.002**	0.999	0.364
Body surface area, kg/m^2^	1.76 ± 0.24	1.73 ± 0.25	1.74 ± 2.24	1.78 ± 0.23	1.000	0.998	0.998
LV end diastolic volume	138 ± 41	124 ± 39	97 ± 45	138 ± 39	1.000	1.000	**0.029**
LV end systolic volume	52 ± 22	41 ± 18	42 ± 25	46 ± 20	0.678	0.954	0.999
LV-EF	63 ± 7	67 ± 5	64 ± 11	67 ± 7	0.382	0.998	0.952
LV-Mass	112 ± 31	97 ± 35	90 ± 19	108 ± 34	0.999	0.933	0.297
LV end diastolic volume indexed	77 ± 20	71 ± 18	54 ± 23	77 ± 23	1.000	1.000	**0.006**
LV end systolic volume indexed	29 ± 19	23 ± 8	24 ± 13	26 ± 7	0.587	0.930	0.999
LV-Mass Index	63 ± 12	56 ± 15	51 ± 8	63 ± 19	1.000	0.701	0.141
RV end diastolic volume	257 ± 84	235 ± 92	297 ± 113	153 ± 32	**< 0.001**	**0.026**	**0.007**
RV end systolic volume	134 ± 63	103 ± 48	160 ± 84	66 ± 16	**< 0.001**	**0.07**	**< 0.001**
RV-EF	49 ± 9	57 ± 6	48 ± 13	57 ± 6	**< 0.001**	1.000	0.248
RV end diastolic volume indexed	145 ± 43	134 ± 40	169 ± 56	85 ± 12	**< 0.001**	**0.002**	**0.001**
RV end systolic volume indexed	76 ± 33	58 ± 21	91 ± 42	37 ± 8	**< 0.001**	**0.012**	**0.010**
							
RV Displacement, mm (± SD)	16 ± 4	30 ± 4	18 ± 3	26 ± 3	**< 0.001**	**0.026**	**< 0.001**

**Figure 2:**
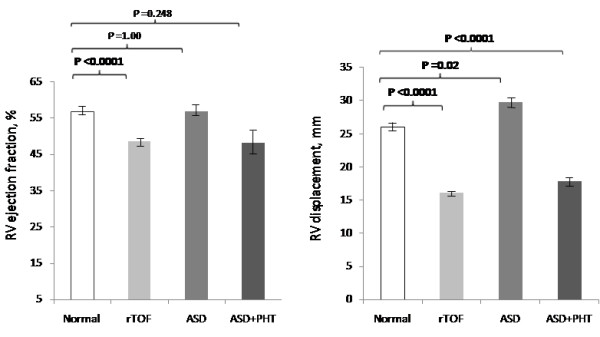
**RV ejection fraction and RV displacement**. Mean RV ejection fraction (± standard error) of the three patient groups compared to normal. RV ejection fraction in rTOF was significantly reduced compared to normal controls. There were no significant differences between the ASD groups and controls. Mean RV displacement (± standard error) in the three patient groups compared to normal. The rTOF and ASD with PHT groups were significantly lower compared to normal. In contrast, ASD without PHT were significantly higher than normal.

There were some differences between the four groups with respect to RV volumes and ejection fraction, as follows. The controls had significantly lower indexed RV volumes and higher ejection fractions relative to the volume and pressure overloaded rTOF (table 2 and figure 2). The RV end-diastolic volumes were significantly higher in the ASD groups (Table 2) but there was no difference in ejection fraction between the ASD groups and controls (Figure 2). Although the mean ejection fraction in the ASD with PHT group was similar to that of the rTOF group, the difference compared to the control group did not reach statistical significance. There were 2 outliers with higher than expected ejection fractions (62 and 75%) which may have contributed to the lack of significant difference.

### RV Displacement

No significant difference in RV displacement was seen between males and females in the control group, 26 ± 4 vs 27 ± 4mm, p = 0.325.

The mean RV displacement was significantly higher in the control group relative to rTOF and ASD with pulmonary hypertension groups but significantly lower than that observed in the ASD without pulmonary hypertension group (Table 2 and figure 2).

**Figure 3:**
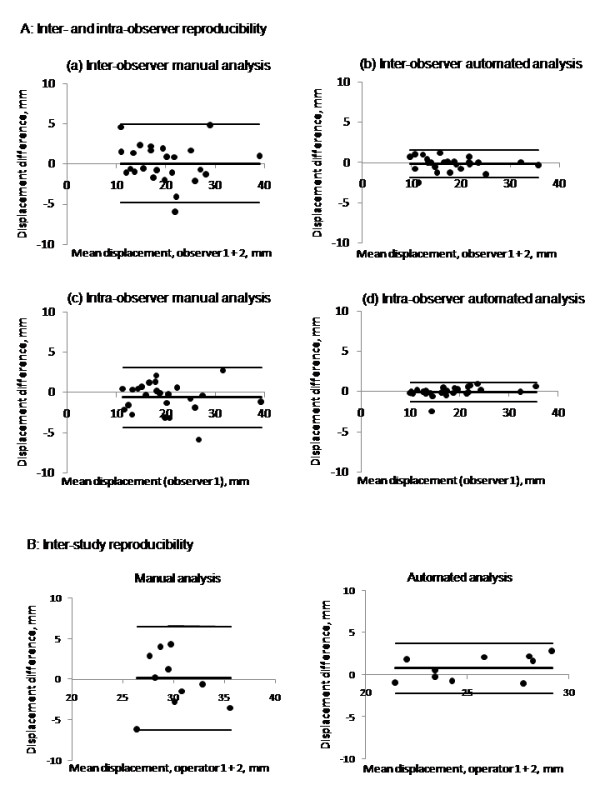
**A and B: A (a-d). Inter- and intra-observer reproducibility of the manual and automated analyses**. Bland-Altman plots showing inter-observer and intra-observer reproducibility of RV displacements by the manual (a and c) and automated (b and d) techniques. The automated analysis showed closer 95% limits of agreement than manual analysis. B. Inter-study reproducibility of RV displacement Bland-Altman plots showing inter-study reproducibility with closer 95% limits of agreement when analysed with the automated technique compared with the manual technique.

The measurement of RV displacement with both the manual and automated techniques proved to be highly reproducible with an inter-observer bias of -0.1 mm for the automated technique and 0.1 mm for the manual technique, but the standard deviation for the automated technique was substantially lower with correspondingly narrower 95% limits of agreement (automated versus manual, bias ± 95% limits of agreement: -0.1 ± 1.6 mm versus 0.1 ± 4.9 mm; Figure 3A (a-b). Agreement was good between automated and manual readings with an intra-class coefficient of correlation (ICC) of 0.87 (P < 0.001). There was also a good degree of intra-observer repeatability in the measurements for both the automated (bias ± 95% limits of agreement of -0.01 ± 1.2 mm; ICC 0.993, P < 0.001) and manual techniques (bias ± 95% limits of agreement of -0.6 ± 3.7 mm; ICC 0.970, P < 0.001). Again, the 95% limits of agreement were tighter for the automated method suggesting higher precision, Figure 3A(c-d). There was also good inter-study repeatability for the automated technique with a bias ± 95% limits of agreement of 0.7 mm ± 2.7 mm (Figure 3B) versus -0.4 ± 6.7 for the manual technique. The inter-study variability was therefore greater than the intra-observer variability in measurements.

**Figure 4:**
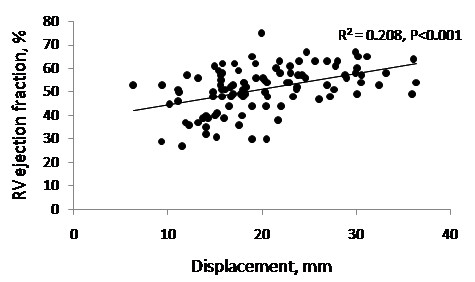
**RV displacement and RV ejection fraction**. There was a weak but significant correlation between RV displacement and RV ejection fraction.

As anticipated, there was a weak but nonetheless significant positive correlation between RV-EF and RV-displacement (Figure 4) reflecting the fact that both are assessing aspects of right ventricular function (r = 0.456, P < 0.001).

## Discussion

The early detection of changes in RV systolic function is of potential clinical value, particularly in the management of patients with congenital heart disease [11] but also increasingly in those with acquired pathology where it has been found to have prognostic implications [12-14]. This requires reproducible and readily implemented techniques for clinical assessment of RV contractile function, enabling changes to be identified with confidence on serial follow up. CMR is widely used for the evaluation of congenital heart disease. However, measurements of RV ejection fraction may be a relatively insensitive method for detecting change of RV systolic function with disease [15,16].

Our data demonstrates the use of a novel CMR tagging technique for the assessment of RV long axis function. Grid or harmonic tagging techniques that have been applied to the left ventricle do not translate readily to the right ventricle owing to its thin walls and complex geometry [3]. Our technique was quick to acquire, in a single additional breath-hold, and to analyse. It recorded differences of RV performance between groups that were not apparent between the measurements of RV ejection fraction. Mean RV displacement was higher in the control (26 ± 3 mm) than in rTOF (16 ± 4 mm) and ASD with pulmonary hypertension (18 ± 3 mm) groups but lower than in the normotensive ASD group (30 ± 4 mm), P < 0.001.

While RV ejection fraction did not differ significantly between the ASD group and the controls, the tagging technique showed less long axis displacement in the ASD group with PHT and more long axis displacement in the ASD group without PHT. This supports the ability of the technique to distinguish ventricular performance between groups of patients with pressure and/or volume loading of the RV, although the previous cardiac surgery, as in the rTOF group, would also be likely to affect the measurements.

Our method of assessing RV long axis function is in principle analogous to the evaluation of tricuspid annular plane systolic excursion (TAPSE) [17]. TAPSE has been shown previously to correlate well with RV ejection fraction by radionuclide angiography in normal subjects and in patients with ischaemic heart disease [17]. This has not been shown in congenital heart disease as very few studies have been undertaken so far in this population of patients. However, the application of the technique of TAPSE is dependent on acoustic access and the angle of insonation [6], and is not feasible in all patients, particularly after heart surgery or when the RV is dilated. Comparable regions and angles of insonation for TAPSE analysis may be hard to achieve between different operators, although this has not to our knowledge been formally investigated. CMR does not suffer such limitations and complete four chamber views of the heart can be aligned relative to specified anatomical landmarks. We took the mid-point of the mitral valve, the point of angulation of the RV free wall adjacent to the cardiophrenic angle and the apex of the left ventricle as the three points that defined our four chamber plane.

A previous study has attempted to quantify RV long-axis function with CMR. It relied on a manually located point on the tricuspid annulus whose motion was followed relative to the RV apex [18]. This technique introduces potential sources of variation between observers or studies. Another approach used 3D-modeling to estimate the distance traversed during systole by a point at the junction of the RV-free wall and tricuspid annulus [19]. We attempted to optimize our reproducibility by tag placement and automated analysis. The analysis only requires the user to identify the tag line on the image in the first frame (end-diastole) and this is then automatically tracked throughout the cardiac cycle [9]. There is no need to define additional reference points or even to identify end systole. The automated analysis technique achieved narrow inter-observer 95% limits of agreement when compared with the manual technique (± 1.6 mm versus ± 4.9) - a greater than three-fold improvement in precision. The gains in intra-observer reproducibility were of a similar magnitude. The intra- and inter-observer reproducibility we obtained with our manual approach was comparable to that obtained in previous CMR studies using equivalent manual techniques [18]. Furthermore, the reproducibility of the manual technique was comparable to that reported in the echo literature [20,21]. The automated analysis took less than 2 minutes to complete and so improved precision substantially with little cost in terms of time.

With a view to serial follow up, the automated approach markedly improved inter-study reproducibility (automated versus manual 95% limits of agreement: ± 2.7 mm versus ± 6.7 mm). This greater reproducibility could serve to reduce sample size requirements if the technique were to be used in clinical trials to assess the impact of therapeutic interventions on right ventricular function. Our data provide the basis for sample size and power calculation for future studies in which RV contractile function may be an end-point.

In keeping with previous studies, we found only a moderate but nonetheless statistically significant correlation between RV displacement measured by the automated CMR technique and RV ejection fraction [18,19]. This is not unexpected as although the two parameters both assess RV systolic performance, RV ejection fraction is a composite of radial and long axis RV myocardial function, LV function as it affects curvature of the septum, and of RV end diastolic volume. As in our study, Konstam *et al *found that RV ejection fraction in ASD patients with pulmonary hypertension (pressure and volume overload) is similar to that in controls, but impaired in a group with pulmonary hypertension without an ASD (pressure but not volume overload), with preservation of ejection fraction in the former group [22].

The incomplete correspondence of RV ejection fraction and measurements of RV displacement may also be accounted for to an extent by measurement errors. While measurements of RV ejection by CMR in healthy individuals have shown good reproducibility, the approach may be less reproducible with disease. Grothues *et al *found that while the inter-study coefficient of variation for RV ejection fraction was 4.3% for patients with normal ventricles, but rose to 10.4% for patients with congestive cardiac failure and 10.0% for patients with left ventricular hypertrophy [23]. With disease, the endocardial border of the relatively trabeculated right ventricle can be increasingly difficult to delineate giving rise to problems with accurately demarcating end-diastolic and end-systolic volumes [24]. As EF is the ratio of the difference in end-diastolic and end-systolic volumes to end-diastolic volumes, errors in the measurement of either of these parameters can summate, increasing the error in the calculation of EF. Our automated technique for the assessment of RV displacement however only requires one measurement in one dimension in a single acquisition, minimising the problem of measurement error.

### Limitations

Our study assessed RV performance at one time point. We did not perform serial follow up of patients to prospectively assess the ability of the novel tagging technique to identify deteriorations in RV function over time. However, the technique was highly reproducible and the differences identified between the pathological groups were far greater in magnitude than the variation resulting from measurement error. Further prospective studies are required to evaluate the utility of this technique in clinical practice.

It is a limitation that we did not prospectively acquire TAPSE measurements by echocardiography in the same patients. When we attempted to collect echocardiographic data retrospectively, we found it to have been sporadic, of variable quality and inadequate for meaningful for comparison with the prospectively acquired CMR data.

A further limitation of the study is that the temporal and spatial resolutions are limited by the need to acquire the data within the duration of a single breath-hold. However, the multi resolution image registration has been shown [9] to provide sub-pixel tracking of the tagged plane. Furthermore, the temporal resolution captures the principle motion of the cycle and therefore smooth interpolation of the tracked plane between time points enables analysis on a finer timescale.

## Conclusions

Measurements of RV long axis displacement by CMR tagging showed more significant differences between the groups studied than did RV-EF. It was reproducible, quick and easy to apply and analyse. Further work is needed to establish CMR normal ranges for RV displacement and to prospectively evaluate the utility of the technique for the serial follow up of patients at risk of RV dysfunction.

## Competing interests

GZ Yang is a director of Cardiovascular Imaging Solutions. None of the other authors have any potential competing interest.

## Authors' contributions

SC, PK, and JK contributed to the conception and design of the study. Data was acquired by SC and RW. Analysis and interpretation of the data, and drafting of the manuscript were performed by SC, JK, TI and PK. The design of and software analysis of the tagging sequence were done by AW and JK. All authors contributed to critical review, intellectual content and final approval of the manuscript.
